# Identification of UDP-Glucuronosyltransferase 2B15 (UGT2B15) as a Target for IGF1 and Insulin Action

**DOI:** 10.3390/cells11101627

**Published:** 2022-05-12

**Authors:** Rive Sarfstein, Karthik Nagaraj, Shivang Parikh, Carmit Levy, Zvi Laron, Dafna Benayahu, Haim Werner

**Affiliations:** 1 Department of Human Molecular Genetics and Biochemistry, Sackler School of Medicine, Tel Aviv University, Tel Aviv 69978, Israel; rives@tauex.tau.ac.il (R.S.); mailkartz@gmail.com (K.N.); barodablaze117@gmail.com (S.P.); carmitlevy@post.tau.ac.il (C.L.); 2 Endocrinology and Diabetes Research Unit, Schneider Children’s Medical Center, Petah Tikva 49292, Israel; laronz@clalit.org.il; 3 Department of Cell and Developmental Biology, Sackler School of Medicine, Tel Aviv University, Tel Aviv 69978, Israel; dafnab@tauex.tau.ac.il

**Keywords:** insulin-like growth factor-1 (IGF1), growth hormone receptor, UGT2B15, Laron syndrome, p53

## Abstract

Normal growth and development in mammals are tightly controlled by numerous genetic factors and metabolic conditions. The growth hormone (GH)-insulin-like growth factor-1 (IGF1) hormonal axis is a key player in the regulation of these processes. Dysregulation of the GH-IGF1 endocrine system is linked to a number of pathologies, ranging from growth deficits to cancer. Laron syndrome (LS) is a type of dwarfism that results from mutation of the GH receptor (*GHR*) gene, leading to GH resistance and short stature as well as a number of metabolic abnormalities. Of major clinical relevance, epidemiological studies have shown that LS patients do not develop cancer. While the mechanisms associated with cancer protection in LS have not yet been elucidated, genomic analyses have identified a series of metabolic genes that are over-represented in LS patients. We hypothesized that these genes might constitute novel targets for IGF1 action. With a fold-change of 11.09, UDP-glucuronosyltransferase 2B15 (*UGT2B15*) was the top up-regulated gene in LS. The *UGT2B15* gene codes for an enzyme that converts xenobiotic substances into lipophilic compounds and thereby facilitates their clearance from the body. We investigated the regulation of *UGT2B15* gene expression by IGF1 and insulin. Both hormones inhibited UGT2B15 mRNA levels in endometrial and breast cancer cell lines. Regulation of UGT2B15 protein levels by IGF1/insulin, however, was more complex and not always correlated with mRNA levels. Furthermore, *UGT2B15* expression was dependent on p53 status. Thus, UGT2B15 mRNA levels were higher in cell lines expressing a wild-type p53 compared to cells containing a mutated p53. Animal studies confirmed an inverse correlation between UGT2B15 and p53 levels. In summary, increased UGT2B15 levels in LS might confer upon patient’s protection from genotoxic damage.

## 1. Introduction

The growth hormone (GH)-insulin-like growth factor-1 (IGF1) endocrine system is a major regulator of basic metabolic and developmental processes throughout life [1,2,3]. Among other physiological actions, the GH-IGF1 axis plays an important role in the growth spurt that embodies the pubertal phase [4]. While levels of GH and IGF1 are reduced in elderly people, clinical and epidemiological data support the view that the GH-IGF1 system also exerts important hormonal activities during aging [5]. IGF1 has been identified as a progression factor during the cell cycle, allowing cells to traverse the entire cycle in the sole presence of sub-physiological concentrations of IGF1 [6,7,8].

In agreement with the mitogenic and pro-survival roles of IGF1, studies have shown that elevated levels of IGF1 correlate with an increased risk of developing a number of tumors, including breast, prostate, and colon cancers [9,10,11,12,13]. The impact of low IGF1 concentrations on cancer risk, on the other hand, has not yet been systematically addressed. Congenital IGF1 deficiencies are part of the causes of growth retardation in children [14,15]. These deficiencies may result from a number of molecular defects along the hypothalamic–hypophyseal axis, including mutations of the GH releasing hormone-receptor (*GHRH-R*), *GH*, GH receptor (*GHR*), *IGF1*, acid-labile subunit, and other genes [16,17,18,19].

Laron syndrome (LS), or primary GH insensitivity, constitutes the best-characterized entity under the umbrella of congenital IGF1 deficiencies [20]. LS is caused by mutations and deletions of the *GHR* gene, leading to GH resistance and dwarfism [21,22,23,24]. The distinctive features of LS are short stature (−4 to −10 SDS below median height), typical face, reduced head circumference, obesity, high basal serum GH and low to undetectable serum IGF1. Lack of IGF1 response to the administration of exogenous GH is regarded as a key diagnostic criterion for LS. Independent epidemiological analyses conducted on two cohorts of LS patients revealed that LS is associated with protection from cancer [25,26,27]. While the mechanisms responsible for cancer protection in LS are yet to be identified, the interpretation of epidemiological data is consistent with the notion that homozygous congenital IGF1 deficiency might confer protection against future development of cancer [28].

We have recently conducted genome-wide profiling of LS-derived lymphoblastoids to identify genes and signaling pathways that are over- or under-represented in patients and that might account for cancer protection. Bioinformatic analyses revealed that the top upregulated genes in LS cells were members of the uridine 5′-diphosphate-glucuronosyltransferase (UGT) gene family [29]. Specifically, the levels of UGT2B15 and UGT2B17 mRNAs were ~7–11 fold higher in LS patients than in controls [30,31].

The UGT enzymes are of major importance in the conjugation and subsequent elimination of potentially toxic xenobiotic compounds [32,33]. UGT constitutes a superfamily of enzymes involved in drug detoxification pathways in the mammalian liver [34]. These enzymes display activity towards several classes of xenobiotic substrates, including simple phenolic compounds, flavonoids, antraquinones, and certain drugs and their hydroxylated metabolites [35]. The UGT enzymes inactivate substrates by the addition of the hydrophilic glucuronyl moiety to acceptor molecules [36]. As a result, the polar metabolites can no longer interact with their receptors, leading to metabolite excretion that is facilitated by an increase in water solubility. There are two major classes of UGT enzymes in humans, UGT1 and UGT2, each containing several genes. The UGT2B15 and UGT2B17 enzymes are members of the UGT2 subfamily and display substrate specificity for a group of androgens, including testosterone, dihydrotestosterone (DHT), and DHT metabolites [37,38].

In view of our results on increased expression of UGT2B15 in LS and given the fact that no information exists regarding a potential link between the IGF1/insulin signaling pathway and UGT2B15 in humans, we investigated in the present study the regulation of *UGT2B15* gene expression and action by IGF1 and insulin. Data indicate that both hormones inhibited UGT2B15 mRNA levels in endometrial and breast cancer-derived cell lines. Regulation of UGT2B15 protein levels by IGF1/insulin, however, was more complex and not always correlated with mRNA levels. In addition, *UGT2B15* gene expression was strongly associated with p53 status. Thus, UGT2B15 mRNA levels were higher in cell lines expressing a wild-type p53 compared to cells containing a mutated p53.

Taken together, our data indicate that increased UGT2B15 levels in LS might confer upon patients a protective effect against oxidative and, potentially, genotoxic damage. If substantiated by functional assays, these findings may provide valuable insight into the physiological basis for reduced cancer incidence in LS.

## 2. Materials and Methods

### 2.1. Cell Cultures and Treatments

Epstein-Bar virus (EBV)-immortalized lymphoblastoid cell lines derived from LS patients and healthy controls were obtained from the National Laboratory for the Genetics of Israeli Populations (Tel Aviv University, Tel Aviv, Israel). Lymphoblastoid cell lines were maintained in RPMI-1640 medium supplemented with 10% fetal bovine serum (FBS), 2 mM glutamine, 100 U/mL penicillin, and 100 μg/mL streptomycin. All reagents were purchased from Biological Industries Ltd. (Kibbutz Beit-Haemek, Israel). The uterine serous papillary carcinoma cell lines USPC-1 and USPC-2 were kindly provided by Dr. A. Santin (Yale University School of Medicine, New Haven, CT, USA). The USPC-1 cell line expresses a wild-type p53 and the USPC-2 cell line expresses a p53 null mutant gene. USPC cells were grown in RPMI-1640 medium supplemented with 10% FBS, 2 mM glutamine, 100 U/mL penicillin, 100 μg/mL streptomycin and 5.6 mg/L amphotericin B.

The estrogen receptor (ER) positive breast cancer cell lines MCF7 and T47D were obtained from the American Type Culture Collection (Manassas, VA, USA). MCF7 cells express a wild-type p53, whereas T47D cells express a mutant p53 gene. Cells were maintained in DMEM supplemented with 10% FBS, 2 mM glutamine, 100 U/mL penicillin, 100 μg/mL streptomycin, and 5.6 mg/L amphotericin B. Cells were incubated at 37 °C in a humidified atmosphere containing 5% CO_2_. For hormonal treatments, cells were serum-starved for 24 h, after which they were treated with IGF1 (50 ng/mL; PeproTech Ltd., Rocky Hill, NJ, USA) or insulin (50 ng/mL; Biological Industries Ltd.). All experiments were carried out at least twice.

A selective IGF1R inhibitor (AEW541) was obtained from Novartis Pharma (Basel, Switzerland). AEW541 is a reversible, ATP-competitive phosphorylation inhibitor with high selectivity towards IGF1R over insulin receptor (INSR). AEW541 was used at a dose of 10 μM for 48 h. A selective INSR inhibitor (S961) was obtained from Novartis Pharma. S961 is a biosynthetic INSR antagonist that inhibits cell proliferation and colony formation in breast tumor cells. S961 was used at doses of 100 nM and 1 μM for 2 h.

### 2.2. Real-Time Quantitative Polymerase Chain Reactions (RT-QPCR)

Total RNA was prepared from cell lines using the Trizol reagent (ThermoFisher Scientific, Waltham, MA, USA). Total RNA (2 μg) was reverse transcribed using the Superscript First-Strand Synthesis System for RT-PCR. UGT2B15 and IGF1R mRNA levels were measured by RT-QPCR, using Faststart Universal SYBR Green Master (Roche AG, Basel, Switzerland) and a StepOne Real-Time PCR system (Applied Biosystems, Foster City, CA, USA). Primers are listed in Table 1. Amplifications were carried out after an incubation of 2 min at 50 °C and 10 min at 95 °C, followed by 40 cycles at 95 °C for 15 s, 1 min at 60 °C, and 30 s at 72 °C. The number of PCR cycles to reach the fluorescence threshold was the cycle threshold (Ct). Each cDNA sample was tested in triplicate and mean Ct values are reported. For each reaction, a “no template” sample was included as a negative control. Fold differences were calculated using the 2 − ΔΔCt method [39]. Levels of β-actin or GAPDH mRNA were used for normalization.

### 2.3. Western Blot Analysis

Cells were grown to confluence, pelleted, and lysed in a buffer containing proteases and phosphatases inhibitors (Cell Signaling Technology, Beverly, MA, USA). Samples were electrophoresed through 7.5% or 10% SDS/PAGE and blotted onto nitrocellulose membranes. After blocking with 5% milk, the blots were incubated overnight with the indicated antibodies. Antibodies against IGF1R β-subunit (3027), INSR β-subunit (3025), phospho-IGF1R/INSR (3024), phospho-AKT (9271), AKT (9272), phospho-ERK1/2 (9106) and ERK1/2 (9102) were purchased from Cell Signaling Technology. Anti-heat shock cognate (HSP70) (SC-7298) and anti-GAPDH (G-9) (SC-365062) were from Santa Cruz Biotechnology (Dallas, TX, USA). Antibodies against UGT2B15 were from Abcam (#154864, Cambridge, UK) and Novus Biologicals (NBP2-94747, Littleton, CO, USA). Blots were washed and incubated with the appropriate horseradish peroxidase-conjugated secondary antibody. Proteins were detected using the enhanced chemiluminescence reaction (Westar Supernova, Cyanagen, Bologna, Italy). HSP70 or GAPDH were used as loading controls.

### 2.4. Small-Interfering RNA (siRNA) UGT2B15 Knockdown

For UGT2B15 knockdown, siRNA against human UGT2B15 SMARTpool (L-002000-00-0005) and non-targeting (NT) pool (D-001810-10-05) (Dharmacon Inc., Lafayette, CO, USA) were used. Negative control [non-targeting (NT)] or siRNA against UGT2B15 were transfected using INTERFERin^TM^ (Polyplus Transfection, Illkirch, France). MCF7 and T47D cells were seeded into 6-well plates the day before transfection. Twenty-five nM of siRNA and 8 μL of INTERFERin^TM^ were used for each transfection. UGT2B15 knockdown was tested after 72, 96, 120, and 144 h (according to each cell line’s optimal siRNA concentration and time point) by RT-QPCR or immunoblotting.

### 2.5. Cell Proliferation Assays

To assess the proliferation rate, cells were seeded onto 6-cm plates (5 × 10^4^ cells/well) and, after 24 h, were transfected with siRNA against UGT2B15, or NT, for 72 h (MCF7) or 96 h (T47D) in triplicates. Cells were counted using a Cellometer Auto X4 Cell Counter (Nexcelom Bioscience, Lawrence, MA, USA) before and after transfection.

### 2.6. Co-Immunoprecipitation (Co-IP) Assays

Total lysates (500 μg protein) were diluted in 200 μL IP buffer [1% Triton X-100, 150 mM NaCl, 20 mM Tris buffer (pH 7.5)] in the presence of proteases and phosphatases inhibitors and immunoprecipitated with anti-p53 (SC-126, Santa Cruz Biotechnology) for 18 h at 4 °C. Protein A/G plus agarose beads (SC-20003; Santa Cruz Biotechnology) were added for 2 h at 4 °C. Immunoprecipitates were electrophoresed and immunoblotted with antibodies against p53 or UGT2B15. Total lysates were loaded as input co-IP control for each sample.

### 2.7. Animal Studies

The generation of the p53-KO mouse model was done by crossing p53^flx/flx^ mice, a gift from Dr. Eli Pikarsky (The Hebrew University of Jerusalem, Israel), with FABP4^Cre+^ mice, including a Fabp4 promoter directing expression of Cre recombinase (Jackson Laboratory, Bar Harbor, ME, USA). These FABP4^Cre+^ transgenic mice were used as a Cre-lox tool for the deletion of p53 floxed sequences in white adipose tissue (WAT). The p53-KO in WAT was validated by genotyping. Whole skin tissue was dissected from mice and snap-frozen in liquid nitrogen, followed by homogenization in RIPA buffer containing protease inhibitors (Roche AG) [40]. Detection of p53 and UGT2B15 in p53-KO and wild-type mice WAT from the hypodermal layer of the skin was done as described above.

### 2.8. Statistical Analyses

The significance of the differences between groups was assessed by Student’s *t*-test (two samples, equal variance). Scanning densitometry analyses were evaluated using TINA imaging analysis software. Signal intensities of proteins were normalized to the corresponding HSP70 or GAPDH protein signals. Data are presented as mean ± SEM of two or three independent experiments. *p* values < 0.05 or <0.01 were considered statistically significant.

## 3. Results

### 3.1. Identification of UGT2B15 as a Differentially Expressed Gene in LS

To identify differentially represented genes that may account for cancer protection in LS, a genome-wide profiling was conducted using EBV-immortalized lymphoblastoid cell lines derived from four female patients with LS and four healthy controls of the same ethnicity (Yemen, Iraq, and Iran) and age range (LS, 44.25 ± 6.1 y; controls, 51.75 ± 11.3 y; mean ± SD; *p*-value = 0.29). Thirty-nine annotated genes that were either over- or under-represented in LS compared to controls were identified (with a fold-change difference cutoff > |2| and *p*-value of < 0.05) [29]. Bioinformatic analyses identified a series of metabolic genes that are highly expressed in LS and that may provide a biochemical foundation for cancer evasion in this condition [41,42,43,44]. With a fold-change of 11.09 (LS vs. control), *UGT2B15* was the top up-regulated gene in LS. The expression of an additional UGT gene, *UGT2B17*, was 7.1-fold higher in LS- than in control-derived cells.

**Figure 1:**
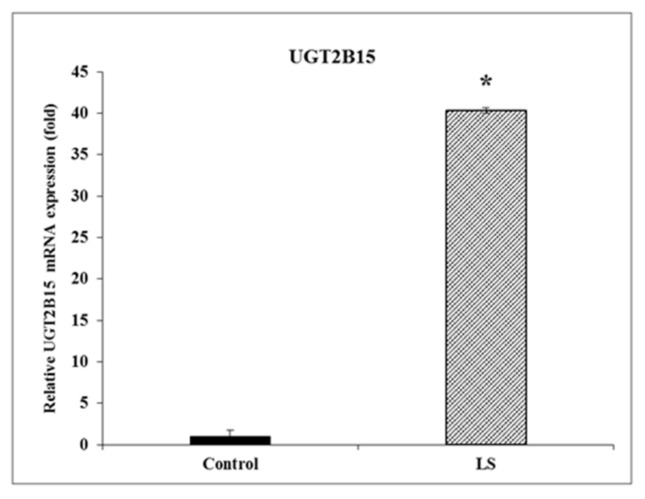
Gene expression of UGT2B15 mRNA in LS patients. Total RNA was obtained from four LS- and four healthy control-derived EBV-immortalized lymphoblastoid cell lines. UGT2B15 mRNA levels were measured by RT-QPCR, as described in Section 2. Results are expressed as UGT2B15 mRNA levels normalized to β-actin mRNA values. A value of 1 was assigned to the UGT2B15 mRNA levels in control cells. Bars denote mean ± SEM. * *p* < 0.01 vs. control cells.

The *UGT* genes code for enzymes that convert xenobiotic and endobiotic substances into lipophilic compounds and thereby facilitate clearance from the body as part of a liver detoxification system. Given the important role of these enzymes in cancer pathology, we hypothesized that UGT2B15 and UGT2B17 are novel targets for inhibitory regulation by IGF1. Genomic data were validated by RT-QPCR, which revealed a 40-fold increase in UGT2B15 mRNA levels in LS, compared to control, cells (Figure 1).

### 3.2. Identification of UGT2B15 as a Target for Inhibitory Regulation by IGF1 and Insulin in Endometrial Cancer Cells

**Figure 2:**
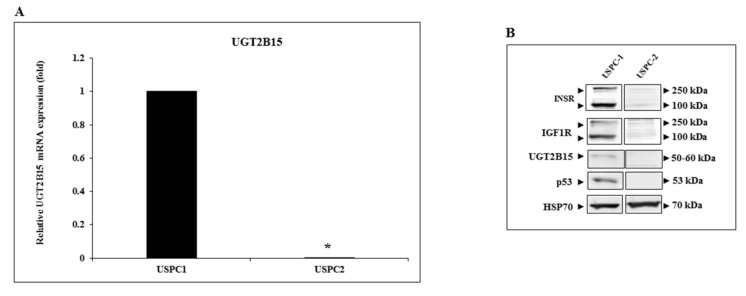
Expression of UGT2B15 in endometrial cancer cell lines. (**A**) Total RNA was obtained from confluent USPC-1 and USPC-2 endometrial cancer cell lines and UGT2B15 mRNA levels were measured by RT-QPCR. A value of 1 was assigned to the UGT2B15 mRNA levels in USPC-1 cells. * *p* < 0.01 vs. USPC-1 cells. (**B**) Total protein was obtained from confluent USPC-1 and USPC-2 cell lines and UGT2B15, IGF1R, INSR, and p53 protein levels were measured by Western blots. HSP70 levels were assessed as a loading control.

To evaluate our hypothesis that *UGT2B15* is a target for inhibitory regulation by IGF1, we chose to measure UGT2B15 mRNA levels in endometrial cancer cells. The rationale for this selection is the fact that endometrial cancer has been strongly correlated with obesity and diabetes and, furthermore, seems to be affected by circulating insulin/IGF1 levels. In initial experiments, we measured UGT2B15 mRNA levels in the uterine serous papillary carcinoma cell lines USPC-1 and USPC-2. These cells differ in the status of tumor suppressor p53. Thus, whereas USPC-1 cells express a wild-type p53 gene, USPC-2 cells contain a mutant p53 [45]. Results of RT-QPCR indicate that basal UGT2B15 mRNA levels were significantly higher in USPC-1 than in USPC-2 cells (Figure 2A). In agreement with these results, Western blots showed that also UGT2B15 protein levels were markedly higher in USPC-1 cells (Figure 2B).

**Figure 3:**
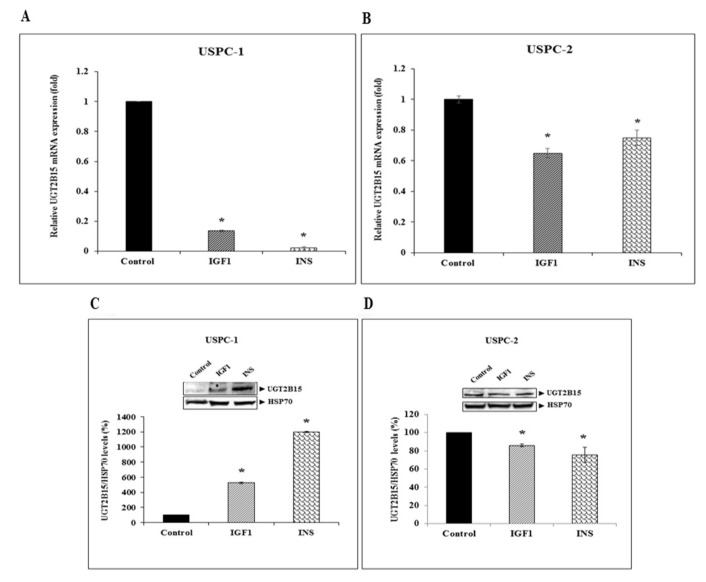
Regulation of *UGT2B15* gene expression by IGF1 and insulin. USPC-1 (**A**) and USPC-2 (**B**) cells were treated with IGF1 or insulin (50 ng/mL) for 24 h, after which total RNA was prepared and UGT2B15 mRNA levels were measured by RT-QPCR. A value of 1 was given to the UGT2B15 mRNA levels in control, untreated cells. * *p* < 0.01 vs. respective control cells. (**C**,**D**) Western blot analysis of hormone-treated USPC-1 and USPC-2 cells. Cells were treated with IGF1 or insulin for 24-h, after which total protein was prepared and UGT2B15 levels were measured by Western blots. Bar graphs represent UGT2B15 levels normalized to the corresponding HSP70 values. Values on the y-axis represent arbitrary units of absorbance. A value of 100% was given to control, untreated cells.

Next, serum-starved cells were treated with IGF1 or insulin (50 ng/mL) for 24 h, after which UGT2B15 mRNA levels were measured. Hormonal treatment led to a marked reduction in UGT2B15 mRNA levels in USPC-1 cells. Thus, IGF1 treatment reduced UGT2B15 mRNA levels by 87%, whereas insulin led to a 98% reduction (Figure 3A). IGF1 and insulin had a reduced effect in USPC-2 cells, in which basal UGT2B15 levels were extremely low (Figure 3B). Of interest, IGF1 and insulin enhanced UGT2B15 protein levels by 5-fold and 12-fold, respectively, in USPC-1 cells (Figure 3C), whereas in USPC-2 cells, both hormones had a small but significant inhibitory effect (Figure 3D).

### 3.3. Regulation of UGT2B15 Gene Expression by IGF1 and Insulin in Breast Cancer Cells

**Figure 4:**
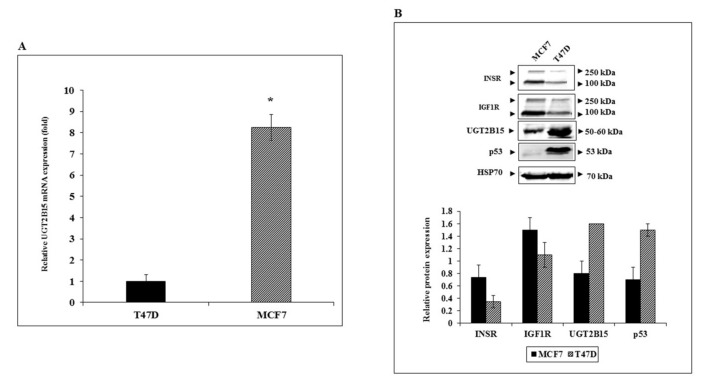
Expression of UGT2B15 in breast cancer cell lines. (**A**) Total RNA was obtained from confluent T47D and MCF7 cells and UGT2B15 mRNA levels were measured by RT-QPCR. A value of 1 was assigned to the UGT2B15 mRNA levels in T47D cells. * *p* < 0.01 vs. T47D cells. (**B**) Total protein was obtained from confluent T47D and MCF7 cell lines and UGT2B15, IGF1R, INSR, and p53 protein levels were measured by Western blots. Relative protein levels are expressed as protein levels normalized to the corresponding HSP70 value. Results of a representative experiment are shown, repeated three times with similar results.

To better characterize the regulation of *UGT2B15* gene expression in breast cancer cells, basal UGT2B15 mRNA levels were measured in two human breast cancer-derived cell lines, MCF7 and T47D. Cell lines differ in the status of tumor suppressor p53. Thus, whereas MCF7 cells express a wild-type p53, T47D cells contain a mutant p53 [46]. Results of RT-QPCR indicate that basal UGT2B15 mRNA levels were 8.25-fold higher in MCF7 than in T47D cells (Figure 4A). On the other hand, UGT2B15 protein levels were higher in T47D cells (Figure 4B).

**Figure 5:**
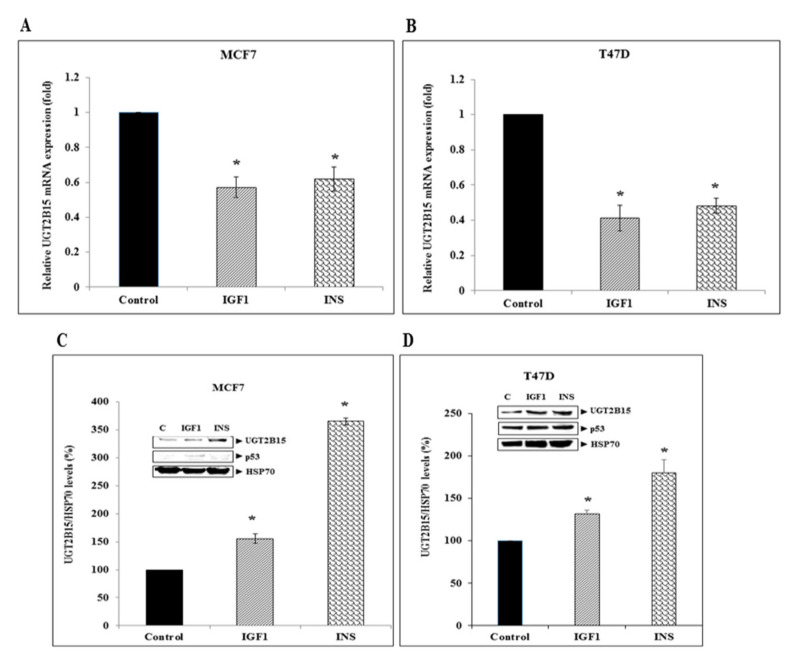
Regulation of *UGT2B15* gene expression by IGF1 and insulin in breast cancer cell lines. MCF7 (**A**) and T47D (**B**) cells were treated with IGF1 or insulin (50 ng/mL) for 24 h, after which total RNA was prepared and UGT2B15 mRNA levels were measured by RT-QPCR. A value of 1 was given to the UGT2B15 mRNA levels in control, untreated cells. * *p* < 0.01 vs. respective control cells. (**C**,**D**) MCF7 and T47D cells were treated with IGF1 or insulin for 24 h, after which total protein was prepared and UGT2B15 and p53 levels were measured by Western blots. Bar graphs represent UGT2B15 levels normalized to the corresponding HSP70 values. A value of 100% was given to control, untreated cells.

Next, the regulation of *UGT2B15* gene expression by IGF1 and insulin was assessed by incubating serum-starved MCF7 and T47D cells with either hormone for 24 h. In both cell lines, IGF1 and insulin reduced UGT2B15 mRNA levels to a similar extent (43–39% reduction in MCF7 cells and 59–52% reduction in T47D) (Figure 5A,B). In contrast, the hormonal treatment increased UGT2B15 protein levels in both breast cancer cell lines (Figure 5C,D).

**Figure 6:**
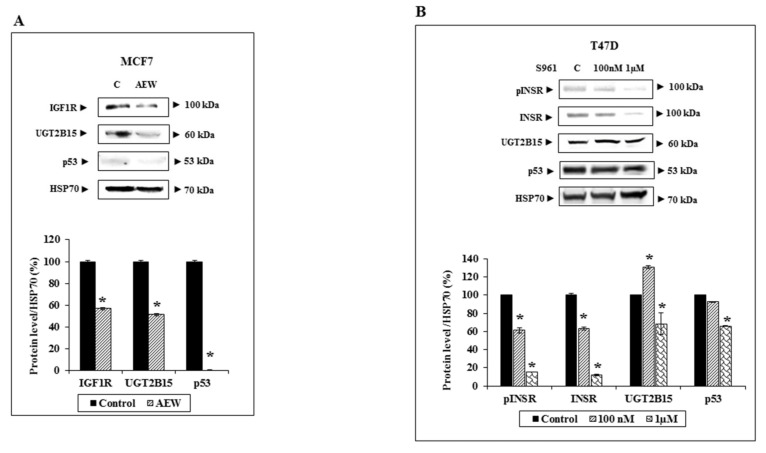
Effect of IGF1R and INSR inhibition on *UGT2B15* gene expression. (**A**) MCF7 cells were treated with the selective IGF1R inhibitor AEW541 (10 mM) for 48 h (or left untreated, C), after which cells were harvested, total protein was prepared, and IGF1R, UGT2B15, and p53 levels were measured by Western blots. HSP70 levels were measured as a loading control. The bar graph denotes UGT2B15 and IGF1R levels in control (solid bars) and AEW541 treated cells (striped bars). (**B**) T47D cells were treated with the INSR inhibitor S961 (100 nM and 1 mM) for 2 h. Cells were then harvested and levels of phospho- and total-INSR, UGT2B15, and p53 levels were measured by Western blots. A value of 100% was given to control, untreated cells. * *p* < 0.01.

To confirm that the effect of IGF1 was mediated via the IGF1R, MCF7 cells were treated with a selective IGF1R inhibitor (AEW541) for 24 h, after which UGT2B15 protein levels were measured by Western blots. As shown in Figure 6A, partial IGF1R silencing led to a parallel reduction in UGT2B15 protein levels. Likewise, p53 levels were diminished upon IGF1R silencing. On the other hand, INSR silencing using S961, a specific INSR antagonist, had a small effect on UGT2B15 levels in T47D cells (Figure 6B). Combined, the data suggest that the effects of IGF1 and insulin on UGT2B15 expression are most probably mediated via the IGF1R.

### 3.4. Paradoxical Effects of UGT2B15 Abrogation on IGF1R Signaling and Biological Action in MCF7 and T47D Cells

To investigate the impact of *UGT2B15* silencing on the expression and activation of downstream IGF1R and INSR signaling mediators, MCF7 and T47D cells were treated with a specific siRNA against UGT2B15 (or NT siRNA for control purposes), after which Western blots were conducted using antibodies against total and phospho ERK1/2 and AKT. Extensive calibration experiments identified optimal doses (25 nM) and treatment periods (72-h for MCF7 and 96-h for T47D cells) (not shown).

**Figure 7:**
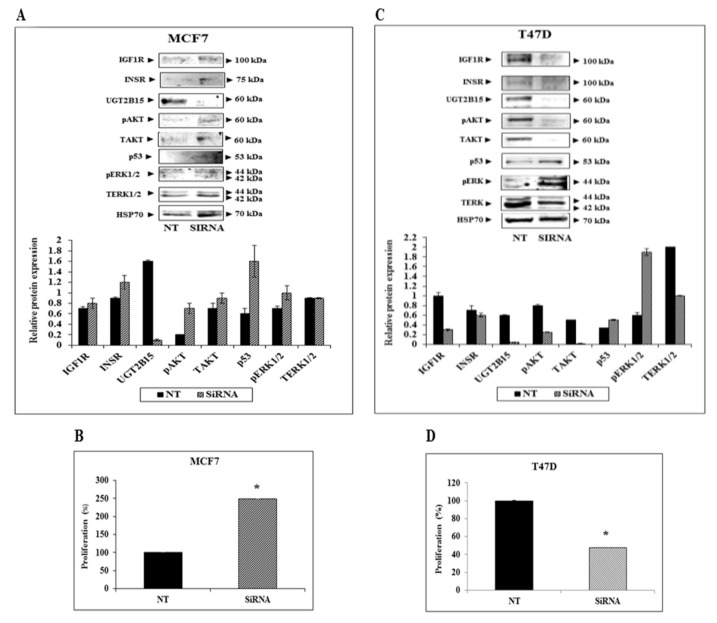
Effect of UGT2B15 abrogation on IGF1R signaling and cellular proliferation. MCF7 (**A**,**B**) and T47D (**C**,**D**) were treated with siRNA against UGT2B15 (or NT for control purposes) for 72-h (MCF7) or 96 h (T47D). At the end of the incubation period, cells were harvested, and the levels of IGF1R, INSR, UGT2B15, phospho- and total- AKT and ERK1/2, and p53 were measured by Western blots. HSP70 levels were measured as a loading control. Relative protein levels are expressed as protein levels normalized to the corresponding HSP70 value. Results of a typical experiment are presented. For cell proliferation measurements, cells were treated with siRNA against UGT2B15 (or NT siRNA) for 72 h (MCF7) or 96 h (T47D). Cells were counted using a cell counter. A value of 100% was given to the cell number in NT-treated (control) cells. * *p* < 0.01 vs. NT-treated cells.

Treatment of MCF7 cells with UGT2B15 siRNA led to increases in IGF1R and INSR protein levels (Figure 7A). In addition, enhanced activation (phosphorylation) of AKT and ERK1/2 was noticed upon UGT2B15 abrogation. Activation of these pathways was associated with a ~2.5-fold increase in proliferation (Figure 7B). On the other hand, UGT2B15 silencing in T47D cells led to a reduction in IGF1R and INSR levels (Figure 7C). In addition, there was a reduction in phospho-AKT and an increase in phospho-ERK levels. Finally, UGT2B15 abrogation in these cells correlated with a 52% decrease in proliferation rate (Figure 7D). Data is consistent with the role of AKT in promoting proliferation.

### 3.5. Co-Immunoprecipitation of UGT2B15 and p53

**Figure 8:**
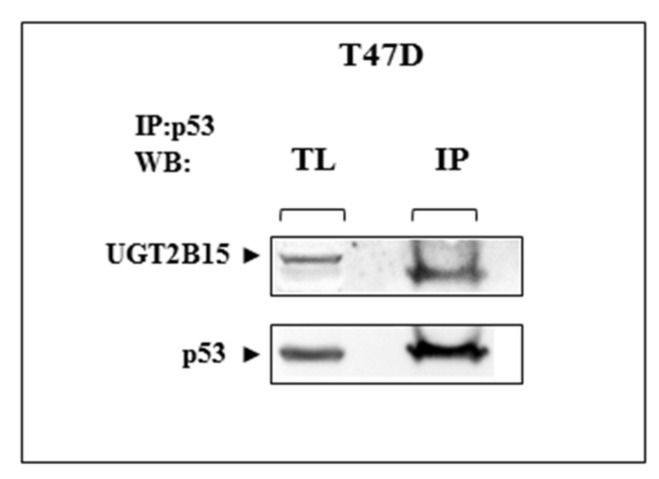
Physical interactions between UGT2B15 and p53. T47D cells were lysed and immunoprecipitated with anti-p53. Precipitates were electrophoresed through 10% SDS-PAGE, blotted onto nitrocellulose filters, and immunoblotted with anti-UGT2B15 or anti-p53. IP, immunoprecipitation; WB, Western blotting; TL, total lysate. Results of a typical experiment repeated twice with similar results are shown.

To investigate a potential physical interaction between UGT2B15 and p53, T47D cells were lysed and immunoprecipitated with anti-p53. The rationale for the use of T47D cells is the fact that these cells express high levels of endogenous p53. Precipitates were electrophoresed through 10% SDS-PAGE, blotted onto nitrocellulose filters, and immunoblotted with anti-UGT2B15 or anti-p53. The results obtained showed that immunoblotting with anti-UGT2B15 identified UGT2B15 protein in p53 immunoprecipitates (Figure 8). These results suggest that the functional interactions between UGT2B15 and p53 are most probably correlated with physical interactions between both proteins. Bioinformatics analysis using the Seek platform (https://seek.princeton.edu/seek/, accessed on 1 March 2022) confirmed a predicted protein-protein interaction between UGT2B15 and p53 (Coexpression score = −0.0975).

### 3.6. Animal Studies

**Figure 9:**
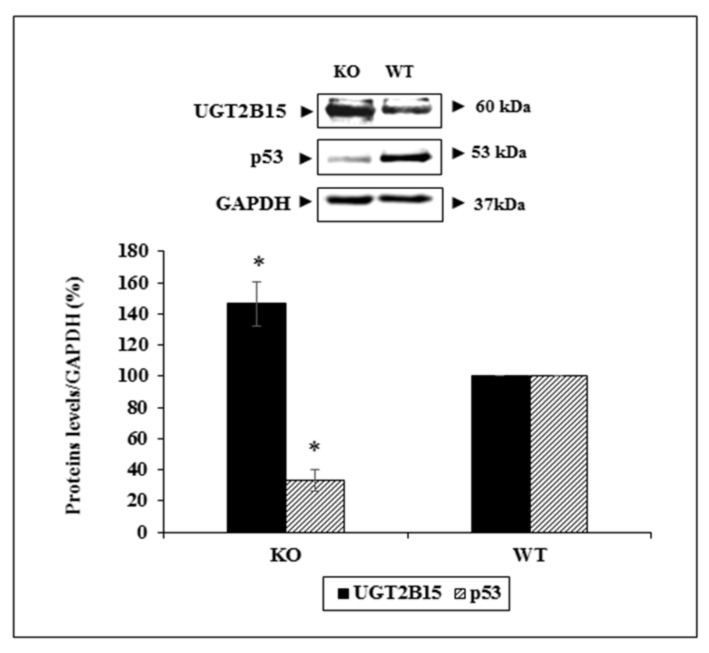
In vivo analysis of interactions between UGT2B15 and p53. Whole skin tissue was obtained from p53-KO and wild-type mice and processed as described in Materials and Methods. Levels of UGT2B15 and p53 were measured by Western blots. GAPDH served as a loading control. Bars represent the mean ± SD of two independent samples. * *p* < 0.01 vs. p53 wild-type mice.

To verify the functional interactions between UGT2B15 and p53 in vivo, we measured the levels of both proteins in WAT of conditional p53-KO mice (and wild-type littermates, for control purposes). As shown in Figure 9, there was a 46.5% increase in UGT2B15 levels in p53-KO cells. In contrast, UGT2B15 levels were markedly reduced in wild-type cells, expressing higher levels of p53. Taken together, these results validate the negative feedback loop between UGT2B15 and p53 as shown in siRNA UGT2B15 assays in T47D cells.

## 4. Discussion

The epidemiological observation that LS patients do not develop cancer is of major clinical relevance [26,27]. These population analyses underscore the central role of the GH-IGF1 system in cancer biology. While high endocrine or tissue IGF1 concentrations and enhanced tumor IGF1R expression are regarded as typical features of malignantly transformed cells, it is of importance to investigate the mechanisms responsible for cancer protection in LS and, probably, other types of congenital IGF1 deficiency [31]. Profiling analyses of LS patients identified sets of genes and signaling pathways that are either over- or under-represented in LS-derived cells compared to age-, gender- and ethnicity-matched controls [29]. Furthermore, bioinformatics analyses allowed us to cluster differentially expressed genes according to their biological functions. Of relevance, approximately 15% of the differentially expressed genes are involved in metabolic activities. Our overall aim was to combine bioinformatics along with biochemical and molecular analyses in order to provide a mechanistic explanation for cancer evasion in LS.

The present study identified the *UGT2B15* gene as a novel target for IGF1 action. *UGT2B15* was the top up-regulated gene in LS (11.09-fold). The closely related *UGT2B17* was also highly expressed in LS cells (7.1-fold), suggesting a similar regulatory pattern. Consistent with its elevated level of expression in LS, a condition associated with diminished IGF1 concentrations, *UGT2B15* gene expression was negatively regulated by IGF1 and insulin in cells containing a wild-type *p53* gene. The fact that *UGT2B15* gene expression was markedly reduced in uterine and breast cancer cell lines expressing a mutant, compared to cell lines containing a wild-type, p53, indicates that expression and, probably, the action of UGT2B15 requires a functional p53 pathway. In fact, animal studies using epidermal keratinocytes derived from p53-KO mice demonstrate an inverse correlation between levels of UGT2B15 and p53. It is unknown, however, whether this feedback loop represents a universal pattern of co-regulation of p53 and UGT2B15. To the best of our knowledge, no previous studies have shown an association between the UGT2B15 enzymatic pathway and p53-mediated genome protection signaling.

The observation that the IGF1/insulin-induced decreases in UGT2B15 mRNA levels were not accompanied by corresponding reductions in UGT2B15 protein levels is, in fact, not unprecedented. There are many biological processes that take place between transcription and translation and that can explain, at least partially, this discrepancy [47]. For example, the different half-lives of mRNAs and proteins, as well as differences between the rates of mRNA transcription and protein translation, can explain the lack of correlation between UGT2B15 mRNA vs. protein basal and hormone-regulated expression.

In addition to the mechanistic aspects of IGF1-dependent *UGT2B15* expression, it is important to understand the physio-pathological implications of enhanced UGT2B15 expression in congenital IGF1 deficiencies. We have previously demonstrated that survival of LS-derived lymphoblastoid cells following oxidative damage was several-fold higher than that of control cells [29]. Enhanced survivability correlated with altered expression of a number of autophagic markers, including LC3β and P62. Given the fact that the *UGT2B15* gene, as well as other genes of the UGT family, code for enzymes responsible for the conversion of endobiotic and xenobiotic substances into excretable metabolites, our results may imply that increased UGT2B15 levels in LS might confer upon these cells a protective effect against oxidative and, potentially, genotoxic damage. If substantiated by functional assays, this finding may provide valuable insight into the physiological basis for reduced cancer in LS. In this context, overexpression of UGT2B isoforms (UGT2B4, 2BT, and 2B15) led to a significant diminution in cellular proliferation in breast and pancreatic cancer cells in association with a decrease in lipids content [48].

The expression and regulation of *UGT2B15* and *UGT2B17* by steroid hormones were recently measured in breast cancer specimens and cell lines [49]. Both enzymes were highly expressed in a subset of ER-a-positive primary breast tumors. In cultured cells, ERa and androgen receptor (AR) transcriptionally regulated *UGT2B15* and *UGT2B17* gene expression via a mechanism that involved the transcription factor FOXA1. In addition, using chromatin immunoprecipitation assays, *UGT2B15* and *UGT2B17* genes were identified as direct targets for AR action [50]. Indeed, AR was required for both basal as well as androgen-regulated expression of UGT genes [51]. Furthermore, *UGT2B15* was identified as a negatively-regulated target gene in castration-resistant prostate cancer and lymph node metastases. Of interest, genetic variants of the *UGT2B15* and *UGT2B17* genes were associated differently with prostate cancer risk [52]. Taken together, these results underscore the importance of UGT2B15/B17 in the context of breast and prostate cancer etiology.

In addition to transcriptional regulation, the *UGT2B15* and *UGT2B17* genes were shown to be regulated by epigenetic mechanisms [53,54]. For example, both genes constitute direct targets of miR-376c action. Thus, *UGT2B15* and *UGT2B17* expression is negatively regulated by the binding of miR-376c to the 3′-untranslated regions of both genes in prostate cancer cells. However, the exact role of microRNAs during cancer progression is yet to be explored. Of interest, the methylation status of prostate cancer cells has a major impact on *UGT2B15* and *UGT2B17* gene expression [55]. Furthermore, hypomethylation of both genes correlated with an increased risk of prostate cancer and may therefore serve as a putative biomarker.

Further support for the emerging role of the UGT gene family in cancer biology was recently provided by a study that identified twenty UGT genes in 33 different cancer types using RNAseq data derived from 9514 cancer patients from the TCGA project [56]. UGT genes exhibited cancer-specific expression profiles and high inter-individual variabilities within cancer cohorts. Data demonstrate widespread expression profiles of UGT genes in human cancers, implying active metabolism of UGT substrates within the tumors. Of notice, tumors were derived not only from drug-metabolizing tissues but also from non-drug-metabolizing tissues. In addition, results identified a correlation between specific intratumoral UGT expression and overall survival. Hence, in situ UGT activity can influence cancer progression and patient survival not only through drug metabolism but also through the inactivation of a series of endogenous bioactive molecules that affect cancer growth.

In conclusion, we identified the *UGT2B15* gene as a downstream target for IGF1 and insulin action. Furthermore, *UGT2B15* expression requires a functional p53, as suggested by the fact that expression levels were largely reduced in uterine and breast cancer cell lines expressing a mutant p53. Animal studies corroborated the inverse correlation between UGT2B15 and p53 expression. Finally, enhanced UGT2B15 levels in LS-derived cells might translate into a broad level of protection from oxidative and genotoxic damage.

## Figures and Tables

**Table 1:** Primer sequences for PCR and product size.

Primers	Sequences (5′–3′)	Product Size
GAPDH-F	GCGCACCGTCAAGGCTGAGAAC	142 bp
GAPDH-R	AATGGTGGTGAAGACGCCAGT	
β-ACTIN-F	CCTGGCACCCAGCACAAT	144 bp
β-ACTIN-R	GGGCCGGACTCGTCATACT	
UGTB15-F	GTGTTACCCCAGAATGACC	101 bp
UGTB15-R	GGGATCCCATGGTAGATCG	

## Data Availability

Not applicable.
